# Magnetic Resonance Imaging of Cardiovascular Fibrosis and Inflammation: From Clinical Practice to Animal Studies and Back

**DOI:** 10.1155/2013/676489

**Published:** 2013-08-22

**Authors:** Adelina Doltra, Philipp Stawowy, Thore Dietrich, Christopher Schneeweis, Eckart Fleck, Sebastian Kelle

**Affiliations:** Department of Internal Medicine/Cardiology, German Heart Institute Berlin, Augustenburger Platz 1, 13353 Berlin, Germany

## Abstract

Late gadolinium enhancement is the technique of choice for detecting myocardial fibrosis. Although this technique is used in a wide range of cardiovascular pathologies, ischemic cardiomyopathy and the workup for myocarditis and other cardiomyopathies make up a significant proportion of the total indications. Multiple studies during the last decade have demonstrated its utility to adequately characterize myocardial tissue and offer diagnostic and prognostic information. Recent T1 mapping techniques aim to overcome the limitations of late gadolinium enhancement to assess diffuse fibrosis. ^19^F magnetic resonance has recently emerged as a promising technique for the assessment of inflammation. In the following review we will discuss the basic aspects of fibrosis assessment with MR and its utility for diagnostic and prognostic evaluation. We will also address the topic of cardiovascular inflammation imaging with ^19^F as a potential new development that may broaden the indications for MR in the future.

## 1. Introduction

Late gadolinium-enhanced (LGE) magnetic resonance (MR) is the technique of choice for assessing fibrosis, as demonstrated by the vast amount of evidence arising from both animal and clinical studies [1, 2]. Its popularity has grown in recent years, and it is now an established technique for the noninvasive diagnosis of cardiovascular diseases. Due to its recognized ability to adequately characterize myocardial tissue, scientific societies currently recommend the use of this technique for the detection of myocardial scar [3]. 

The basic principle of late gadolinium enhancement imaging lies in the delayed washin and washout of gadolinium contrast media in tissue with an increased proportion of extracellular space. It is important to note that since LGE is based on differences in extracellular space in different areas of the myocardium, it is only useful if the disease is localized (myocardial infarction being the most typical example). While that is usually not a problem in ischemic heart disease, it may be an issue in other pathologies in which general fibrosis plays an important role (such as valvular or hypertensive heart disease).

Although the indications for performing an MR exam with LGE cover multiple cardiovascular pathologies, a recent European survey [4] has pointed out that the current main indications for this technique are ischemic cardiomyopathy and the workup for myocarditis and cardiomyopathies (which represent 34.2% and 32.2%, resp., of all examinations). 

In this review article we discuss the basic aspects of fibrosis assessment with late gadolinium enhancement as well as its main applications in the field of ischemic heart disease, myocarditis, and other cardiomyopathies. Finally, we offer an insight into new promising techniques (such as diffuse fibrosis detection with T1 mapping, coronary artery wall imaging, and detection of inflammation with ^19^F) which, although currently mainly restricted to research purposes, may be used in the future in clinical practice.

## 2. Methodology

### 2.1. Gadolinium Contrast Agents

Gadolinium (Gd) is an ion with paramagnetic properties, which is toxic in its unbound state. Therefore, the contrast agents (CAs) consist of a large molecule which comprises a Gd ion and a carrier agent that keeps Gd bound during intravenous injection, while still retaining its imaging properties. Gadolinium CAs are relatively large in size which prevents them entering the intracellular space under normal conditions and are thus extracellular agents. Some CAs have a particularly large molecule (like the albumin-bound CA), remain mainly intravascular with just a small degree of extravasation, and are considered vascular agents [5]. 

Most of the CAs are renally excreted, have a half-life of 2 hours, and are almost completely cleared from the bloodstream after 24 hours. 

### 2.2. Adverse Reactions

Adverse reactions to Gd-based CA are uncommon, although potentially severe. In addition to allergic reactions (which are usually mild), a potential adverse effect of Gd CA is nephrogenic systemic sclerosis (NSF), a rare fibrosing disease involving the skin, subcutaneous tissue, and internal organs that can cause severe disability and even death. The reported cases of this disease involve patients with severe (glomerular filtration rate < 30 mL/min/1.73 m^2^) or end-stage renal failure, which can be acute or chronic, and who received repeated high doses of Gd CA in a short period of time. Although liver transplantation was also considered to be a risk factor for the development of NSF, some reports seem to suggest otherwise [6].

Although the precise cause of this disease is unknown, it is believed that it may be explained by the combination of high CA doses, reduced clearance due to renal failure, use of low stability chelating agents, and the presence of a trigger or predisposing condition [7]. Although few cases have been reported, due to this potential adverse effect the use of Gd CA in high-risk patients should be avoided, and low doses should be used in the few cases in which the benefit of its administration clearly outweighs the risk. The incidence of NSF has markedly decreased in the recent years, probably as a consequence of avoiding the use of Gd in high-risk patients [8].

### 2.3. Assessment of Fibrosis with LGE

#### 2.3.1. Physiopathology

As already discussed, gadolinium contrast medium disperses in the extracellular space without entering myocardial cells. However, in certain pathological conditions the volume of distribution of gadolinium may increase due to the expansion of the extracellular space or to the loss of integrity of the cell membrane (which allows for the accumulation of Gd in the intracellular space). The reasons for this increase in the distribution volume vary according to the underlying pathology: in the case of acute myocardial infarction, it is caused by myocardial edema and myocyte necrosis and lysis [9], while in a chronic infarction normal myocardium is substituted by fibrous scar tissue, which has a larger extracellular space [10]. 

### 2.4. Technical Considerations

**Figure 1:**
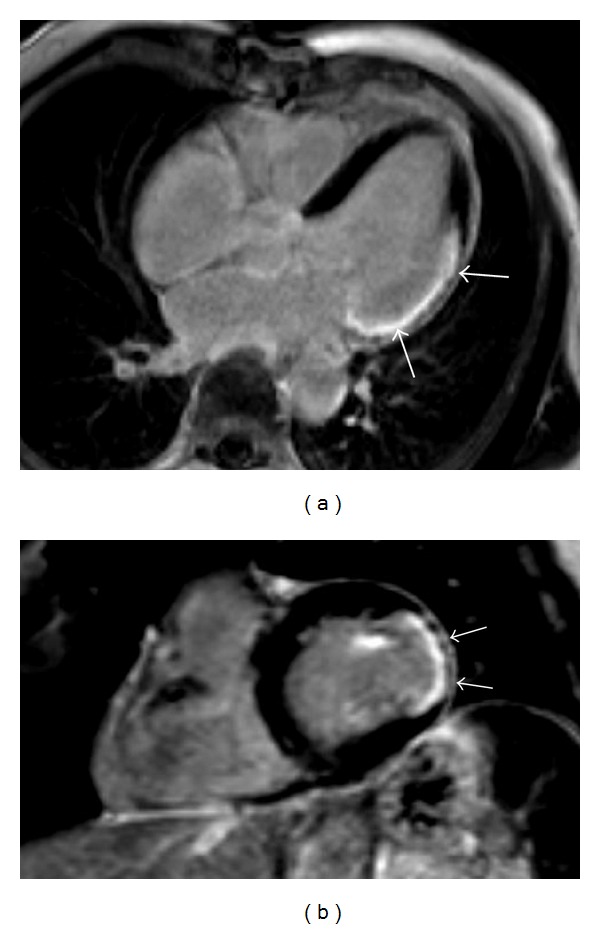
Delayed-enhancement MR images of a patient with chronic lateral myocardial infarction. Both the 3-chamber view (a) and the short-axis view (b) demonstrate the presence of a transmural lateral scar (arrows).

Gd reduces T1 time, which is a tissue-specific parameter. Since every tissue has its own T1 value, this parameter can be used to differentiate between tissues. The MR sequences in which most of the contrast between tissues is due to differences in T1 values are called T1 weighted and are the ones used to obtain images in the LGE technique. In particular, the inversion-recovery gradient-echo sequence used nowadays provides an excellent contrast between normal myocardium and scarring by nulling the signal from normal myocardium (that is to say, it appears “dark” in the image) (Figure 1).

The LGE technique consists of an injection of a 0.1-0.2 mmol/kg bolus of Gd-based CA, followed by T1-weighted acquisition 10–20 minutes after administration of the contrast. The main determinant factor for obtaining good quality images is the correct selection of an adequate inversion time (TI). This parameter is highly variable among patients and physiological conditions, and its correct selection is essential to achieve an effective suppression of normal myocardium (i.e., to obtain a “dark” image in normal myocardium). The TI is selected either visually or by using a Look-Locker sequence, which offers a series of images with variable TI and allows the examiner to pick the one with the best contrast. Phase-sensitive inversion recovery (PSIR) techniques are an alternative to this approach.

### 2.5. Correlation to Histopathology

Hyperenhancement on LGE has a close correlation to histopathologically proven myocardial necrosis, as demonstrated by some studies. In an experimental study, Kim and colleagues [1] demonstrated with ex vivo MRI that the extent of hyperenhancement was the same as the spatial extent of myocardial necrosis at 1 and 3 days after infarction and the scar at 8 weeks. In another experimental study, the best correlation with postmortem evaluation was achieved when using semiautomatic techniques for analysis of scar (in contrast to visual assessment) [11]. Finally, in a clinical study the reproducibility of LGE MR for infarct size determination using quantification software was compared with that of SPECT, demonstrating good clinical reproducibility [2].

## 3. Clinical Applications of LGE

### 3.1. Acute Ischemic Heart Disease

#### 3.1.1. Acute Myocardial Infarction Detection

Some initial animal studies suggested the potential utility of LGE MR in acute myocardial infarction (MI). In a canine model of MI, an increase in the partition coefficient of Gd in the infarcted regions was observed, which was significantly different from noninfarcted regions. This increase in the partition coefficient started 1 min after reperfusion (following 2 hours of coronary occlusion) and persisted until 8 weeks [12, 13]. LGE MR has been shown to be able to accurately detect the presence of an MI, even in the absence of ECG changes or wall motion abnormalities [14]. When compared to SPECT, MR seems to have a higher performance related to a higher spatial resolution, which is of special importance in the case of subendocardial infarctions. In a later study with a dog model of myocardial infarction, both SPECT and MR detected all the segments with nearly (>75%) transmural infarction, but the ability to detect subendocardial infarctions (affecting less than 50% of the myocardial wall) was markedly different between both techniques. Whereas MR identified 92% of segments with subendocardial infarction, only 28% were detected with SPECT [15]. 

In addition to the detection of the infarcted area, MR permits the detection of the ischemic area at risk by combining LGE MR with T2-weighted black blood imaging. Although this area does not demonstrate enhancement, it typically demonstrates a high intensity in T2-weighted imaging. 

#### 3.1.2. Prognostic Value

**Figure 2:**
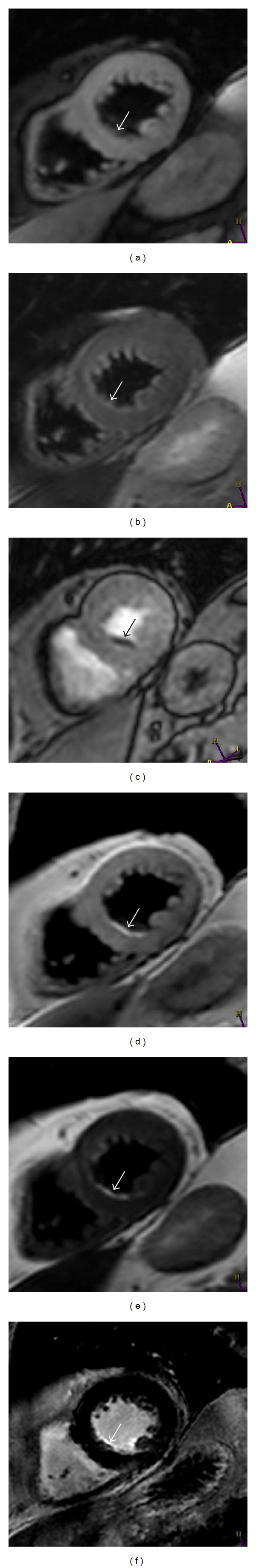
Cardiac MRI at 3.0 Tesla in a patient with coronary artery disease and a history of an old myocardial infarction. At cardiac ultrasound and cine MR imaging no wall motion abnormalities were seen. At T1w ((a) and (d)) and T2w ((b) and (e)) a small subendocardial scar (arrow) which was altered mostly into fat (proven on MR sequences with ((a) and (b)) and without ((d) and (e)) fat suppression) could be demonstrated. There were a small perfusion deficit at rest perfusion (c) and demonstration of a small amount of fibrotic tissue after administration of gadolinium (f).

In addition to its diagnostic utility, the extent of the enhanced myocardium seems to be related to functional parameters at followup. Acute MI mass was demonstrated to be correlated not only with peak troponine I but also with left ventricle (LV) ejection fraction (EF) at followup [16]. Further works have also shown that the acute transmural extent of enhancement of a particular segment is related to its functional improvement and recovery; that is, the larger the extent of hyperenhancement, the lower the likelihood of improvement. Whereas no differences in likelihood of improvement were observed between segments without enhancement and those with up to 25% hyperenhancement relative to the myocardial wall, segments with a higher extent of hyperenhancement had a significantly lower probability of improvement [17, 18] (Figure 2).

#### 3.1.3. Microvascular Obstruction

Another important determinant of prognosis in patients with acute MI is the presence of a microvascular obstruction (MVO), which can also be successfully visualized by MR as an subendocardial area of low signal intensity inside the enhanced region. The assessment of MVO can be performed during the contrast first pass, early (2–5 min) after its administration, or on late gadolinium-enhanced images. It has to be taken into account that the size of MVO decreases over time (i.e., it appears smaller in LGE images), but a lower variability on quantification has been obtained with LGE imaging [19]. The presence of MVO has been associated with a higher rate of adverse cardiovascular events as compared to patients without MVO and remained a negative prognostic marker even after adjustment for infarct size [20].

#### 3.1.4. Infarct Hemorrhage

Lately, there has been a growing interest in the presence of infarct hemorrhage as a strong marker of adverse cardiovascular events and LV remodelling [21]. Although some studies have used T2-weighted imaging for its detection, T2*-weighted sequences are particularly sensitive to the degradation products of hemoglobin. Some animal studies have demonstrated the potential utility of T2*-weighted sequences for hemorrhage assessment [22, 23] although more clinical confirmation studies are needed.

#### 3.1.5. Infarct Peripheral Zone

Finally, the infarct peripheral zone has also been shown to provide prognostic information and can also be detected by LGE MR. In the peripheral zone both viable and nonviable myocytes are present and constitute an arrhythmogenic substrate, whereby the extent of the peripheral zone is a predictor of cardiovascular and all-cause mortality [24]. The peripheral zone possesses lower signal intensity in LGE imaging in comparison to the infarct core, and its detection on MR is based on using different signal intensity thresholds for these two regions. In addition to its prognostic information, scar characterization by LGE MR can also be used to guide ventricular tachycardia ablation in ischemic patients [25]. 

### 3.2. Chronic Ischemic Heart Disease

#### 3.2.1. Differential Diagnosis

**Figure 3:**
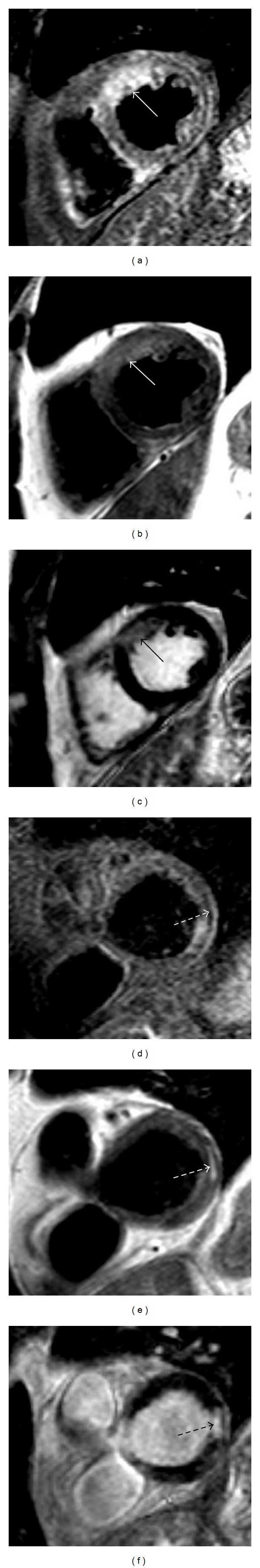
MR imaging in a patient with an acute myocardial infarction (upper row, (a)–(c)) and coincidence of an old myocardial infarction (lower row, (d)-(e)). T2w images with ((a) and (d)) and without ((b) and (e)) fat suppression help to differentiate between the acute phase (edema seen at (a) and (b) (white full) arrow) and chronic stage of an infarction (((d) and (e)), white-dotted arrows). At postcontrast imaging, in the acute phase ((c), black arrow) and chronic stage ((f), black-dotted arrow) infarcted areas demonstrate hyperenhancement, however, with different signal intensities.

Although both acute and chronic MI scars show LGE, the presence of wall thinning and the absence of hyperintensity in T2-weighted imaging suggest chronic MI (Figure 3). In addition to the differentiation between an acute and a chronic MI, it can also be of interest to differentiate between an ischemic cardiomyopathy and a nonischemic cardiomyopathy in a patient that presents with a ventricular dysfunction of unknown origin. A scar that affects the subendocardium and coincides with a coronary territory supports ischemic heart disease, whereas a healthy subendocardium or a noncoronary, “patchy” distribution would indicate a nonischemic cardiomyopathy [26].

#### 3.2.2. Prognostic Value

Similar to acute MI, in chronic ischemic heart disease (IHD) MR can be useful to predict the probability of functional recovery with an inverse relationship between the transmural extent of LGE and the likelihood of functional improvement after revascularization [27]. The percentage of dysfunctional LV without LGE was found to be strongly associated with improvement in EF and global wall-motion score after revascularization. 

On the other hand, the concomitant use of low-dose dobutamine stress MR may offer additional information to that of LGE MR alone, both in terms of functional recovery [28] and to predict clinical outcomes [29]. Both dobutamine inducible wall-motion abnormalities and LGE have been shown to be predictors of hard cardiac events at long-term followup, suggesting an incremental value of these two techniques for the assessment of long-term prognosis [30].

### 3.3. Aorta and Coronary Artery Imaging

In Takayasu arteritis, which usually affects the aorta and its main branches, the presence of delayed enhancement of the artery wall seems to be correlated with inflammatory markers and disease activity [31, 32].

Detection of LGE in the coronary artery wall is also feasible. In a study by our working group, the presence of LGE in the coronary artery wall of all coronary artery disease (CAD) patients could be observed, but it was not seen in any of the healthy subjects [33]. In addition to that, the presence of LGE in the coronary artery wall seems also to correlate with the severity of atherosclerosis as assessed by multislice computed tomography (MSCT) and coronary angiography [34]. 

**Figure 4:**
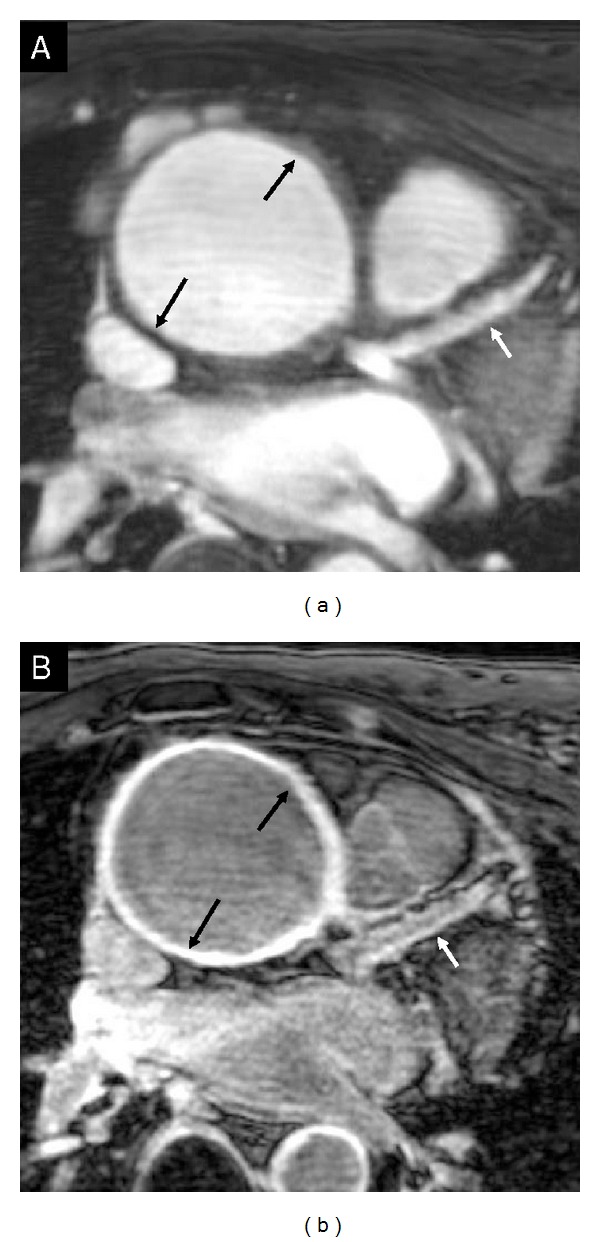
Cross-sectional view of the ascending aorta (black arrows) and longitudinal view of the proximal part of the left anterior descending coronary artery (white arrows) with T2Prep (a) and postgadolinium enhancement MR imaging (b). There is strong vessel wall enhancement after administration of contrast on both the aorta and the coronary artery.

However, the exact significance of the presence of enhancement on the coronary wall remains unclear, as demonstrated by the fact that LGE of the coronary artery wall has also been found in patients with Takayasu arteritis, with a similar proportion to that of CAD patients (Figure 4). The LGE of the coronary wall could, then, be secondary to both inflammation and/or fibrosis [35].

**Figure 5:**
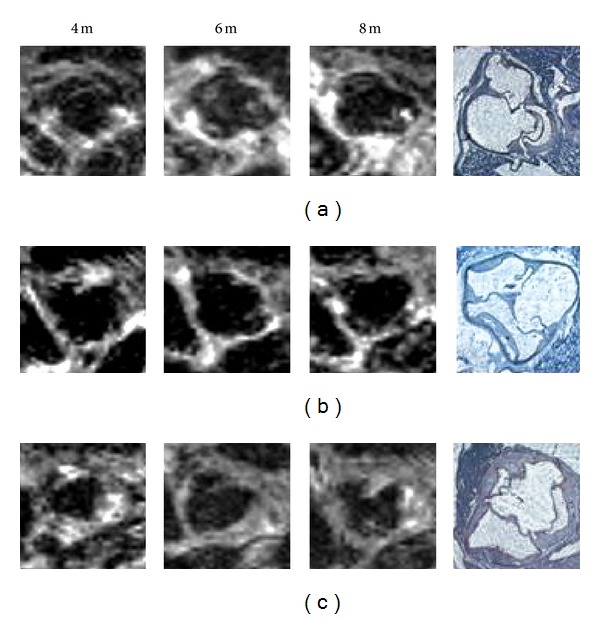
Contrast-enhanced 7 T MRT images of the aorta from ApoE-deficient rats. Three different groups (fat-rich diet with ezetimibe during 8 months (a), fat-rich diet during 8 months and ezetimibe the last 4 months (b), and fat-rich diet without ezetimibe during 8 months (c)) were studied at 4, 6, and 8 months and compared with histology at 8 months. The aortic wall thickness increased with a fat-rich diet, whereas this effect was inhibited with ezetimibe (modified from doctoral dissertation of Dr. Weyers, Charité Faculty of Medicine, Berlin; unpublished data).

Finally, the potential utility of Gd-enhanced MR for differentiating between atherosclerotic coronary plaque types has also been demonstrated [36]. In addition to that, some animal data at high-field MR have shown the potential utility of this technique for the followup of atherosclerotic disease [37] (Figure 5).

The assessment of LGE in the coronary wall currently remains limited to research studies and is not yet used in daily clinical practice. 

### 3.4. Myocarditis

**Figure 6:**
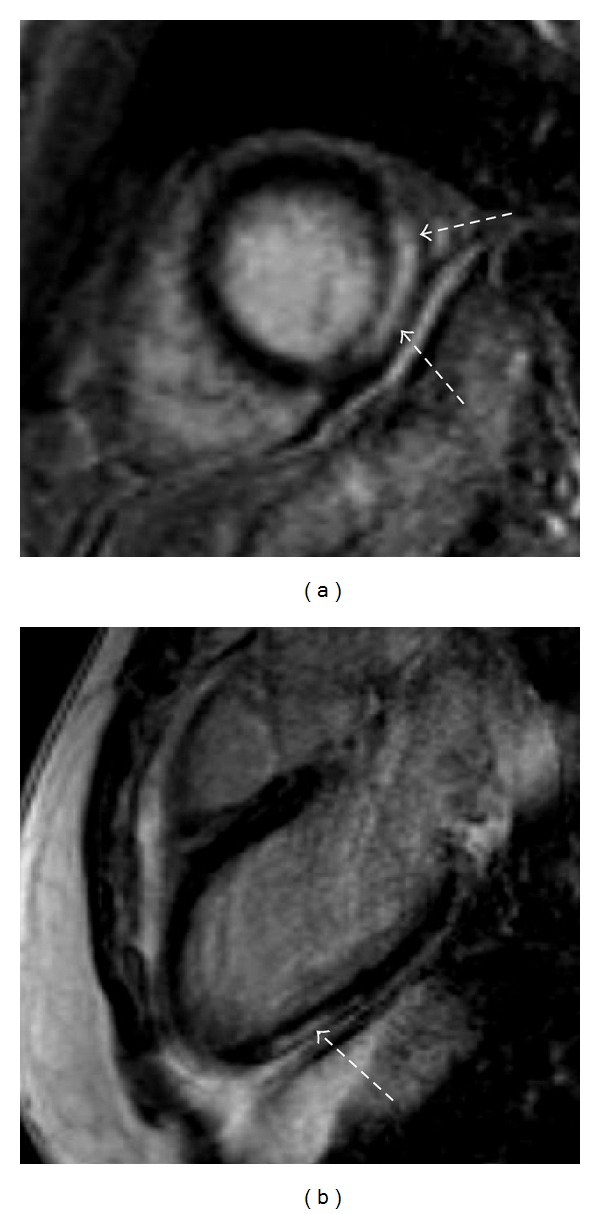
LGE imaging in a patient with a history of myocarditis. There are typical patterns of hyperenhanced areas (dotted arrows), suggesting fibrotic tissue in the mid-inferolateral segments in the short-axis orientation (a) and apical inferior in the two-chamber-view orientation (b).

MR is a useful technique for the diagnosis of myocarditis. The typical pattern of LGE in myocarditis is the presence of patchy enhancement with a subepicardial localization, usually affecting the inferolateral segments and, less frequently, the anteroseptal region [38] (Figure 6). 

The hyperenhanced areas correlate also with histologic active inflammation. In a study with both MR and biopsy, histologically active myocarditis was found in 19 out of 21 patients with biopsy coincident with the area of LGE, but in only one patient in which the biopsy region did not coincide [38]. In addition to LGE, other MR sequences (such as early gadolinium enhancement and edema visualization with T2-weighted imaging) can also be of diagnostic utility. A higher diagnostic performance has been demonstrated when any two of the aforementioned sequences were positive as compared with LGE alone [39]. In another work, MR alone diagnosed 80% of patients with chest pain, positive necrosis enzymes, and absence of CAD although the diagnostic accuracy was improved when MR was combined with endomyocardial biopsy (95% of patients were diagnosed) [40]. The combination of LGE with other MR sequences can also be helpful for differentiating myocarditis from an acute MI [41], and to discriminate between acute and chronic myocarditis [42].

LGE MR can also offer prognostic information in patients with myocarditis. In a recent work including more than 200 patients with biopsy-proven myocarditis and MR, LGE was the best independent predictor of both all-cause and cardiac mortality, with a hazard ratio superior to that of functional class, EF, or LV volumes [43].

### 3.5. Other Nonischemic Cardiomyopathies

The LGE method is also useful for the evaluation of other nonischemic cardiomyopathies. Although different patterns of LGE have been described for various nonischemic pathologies, the key characteristic is that, in contrast to ischemic cardiomyopathy, the subendocardial region is usually not affected. The hyperenhancement regions are usually subepicardial or midventricular and do not correspond to any known coronary territory. In the case of amyloid, the main distinct feature is the difficulty of choosing an optimal TI value to null the myocardium [44].

In addition to its diagnostic utility, the presence of LGE can also offer prognostic information in some entities. In the case of hypertrophic cardiomyopathy, the presence of hyperenhancement has been shown to be an independent predictor of both cardiovascular and global mortality, and of ventricular arrhythmias [45, 46].

### 3.6. Assessment of Fibrosis with T1 Mapping

As it has been described before, although LGE reliably detects localized fibrosis, it is limited in the assessment of diffuse fibrosis. The main reason for this limitation lies in the fact that the LGE technique detects fibrosis on the basis of the difference in signal intensity between an enhanced area and the normal myocardium. As a consequence, no signal intensity difference will be detected if there is a pathology which causes diffuse fibrosis. In order to overcome this limitation of LGE, T1-mapping techniques have been developed which permit the assessment of diffuse fibrosis. This new method allows for evaluation of pathologies which, although associated with a high fibrosis content, cannot be studied with LGE. These T1-mapping techniques permit the direct quantification of the myocardial T1 time. In the setting of an increase in collagen fibers (and thus extracellular space), T1 time decreases after contrast administration. In a recent study with cardiomyopathy patients with endomyocardial biopsy, postcontrast T1 times correlated histologically with fibrosis [47] and were found to be shorter in those patients as compared to healthy controls [48]. 

The modified Look-Locker inversion recovery (MOLLI) sequence is frequently used to measure T1 times, and its reproducibility has been demonstrated [49]. MOLLI T1 mapping has been applied to the study of patients with acute and chronic myocardial infarction (MI), observing different patterns of T1 changes between acute and chronic MI [50]. Regarding the variability of T1 mapping depending on acquisition factors, T1 times vary significantly with field strength (higher at 3 T compared to 1.5 T) and a recent study observed minor variations in T1 time during the cardiac cycle and in myocardial regions (higher in the septum), which should be taken into account when performing T1 measurements [51]. 

Despite promising results, T1 mapping is currently still limited to research purposes and is not yet widely used in clinical practice.

### 3.7. MRI Detection of Inflammation: ^19^F MRI

This technique is based on the utilization of ^19^F to detect inflammation. Because fluorine is only found in the body at very low levels, there is no background signal and it offers excellent specificity [52]. Perfluorocarbons (PFC), which can contain a high concentration of ^19^F, have the advantage of being taken up by the reticuloendothelial cells and, as a consequence, can be used to track these cells. Once they are administered intravenously, PFCs are taken up predominantly by monocytes [53] and transported to inflammatory zones. An alternative approach is in vitro labeling of immunologic cells which are later administered intravenously.

**Figure 7:**
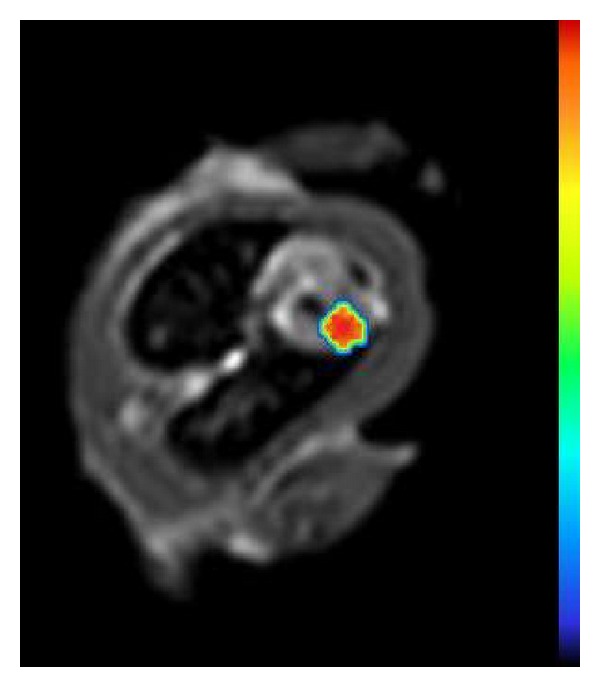
Overlay of an anatomical T2w MR image of a rat thorax (transversal orientation) with the ^19^F signal in false colors. Enrichment of a ^19^F contrast agent is observed in the inflamed area of the heart (doxorubicin-induced myocarditis).

In a study with a murine model of MI, the authors demonstrated the feasibility of visualizing with fluorine MR a infiltration of PFCs in the border zone of the infarcted areas, which corresponded to cells of the monocyte/macrophage system in the histologic analysis [54]. Another study successfully visualized acute rejection in a murine model of heart transplantation [55]. The results of these studies suggest the future utility of this promising technique for imaging inflammation and, even, monitoring treatment (Figure 7), although currently it is not used in daily clinical routine.

## 4. Conclusions

Late gadolinium-enhanced MR is an essential technique for assessing cardiovascular localized fibrosis. In the field of myocarditis and ischemic cardiomyopathy, LGE MRI is a valuable tool that offers diagnostic and prognostic information and can guide therapeutic decision. Although still not widely available in clinical practice, T1 mapping offers the possibility of overcoming the limitations of late gadolinium enhancement by visualizing diffuse fibrosis. Finally, ^19^F MRI is a promising method for imaging cardiovascular inflammation although it is currently still limited to research purposes. The further development of these promising new techniques will expand the application of MR in the future.
